# Unraveled roles of Cav1.2 in proliferation and stemness of ameloblastoma

**DOI:** 10.1186/s13578-022-00873-9

**Published:** 2022-09-03

**Authors:** Shujin Li, Dong-Joon Lee, Hyun-Yi Kim, Jun-Young Kim, Young-Soo Jung, Han-Sung Jung

**Affiliations:** 1 Division in Anatomy and Developmental Biology, Department of Oral Biology, Taste Research Center, Oral Science Research Center, BK21 FOUR Project, Yonsei University College of Dentistry, Seoul, South Korea; 2 NGeneS Inc, Ansan-si, Gyeonggi-do South Korea; 3 Department of Oral & Maxillofacial Surgery, Yonsei University College of Dentistry, Seoul, South Korea

**Keywords:** Ameloblastoma, Proliferation, Stemness, Calcium signaling, Cav1.2

## Abstract

**Background:**

Transcriptome analysis has been known as a functional tool for cancer research recently. Mounting evidence indicated that calcium signaling plays several key roles in cancer progression. Despite numerous studies examining calcium signaling in cancer, calcium signaling studies in ameloblastoma are limited.

**Results:**

In the present study, comparative transcriptome profiling of two representative odontogenic lesions, ameloblastoma and odontogenic keratocyst, revealed that Cav1.2 (*CACNA1C*, an L-type voltage-gated calcium channel) is strongly enriched in ameloblastoma. It was confirmed that the Ca^2+^ influx in ameloblastoma cells is mainly mediated by Cav1.2 through L-type voltage-gated calcium channel agonist and blocking reagent treatment. Overexpression and knockdown of Cav1.2 showed that Cav1.2 is directly involved in the regulation of the nuclear translocation of nuclear factor of activated T cell 1 (NFATc1), which causes cell proliferation. Furthermore, a tumoroid study indicated that Cav1.2-dependent Ca^2+^ entry is also associated with the maintenance of stemness of ameloblastoma cells via the enhancement of Wnt/β-catenin signaling activity.

**Conclusion:**

In conclusion, Cav1.2 regulates the NFATc1 nuclear translocation to enhance ameloblastoma cell proliferation. Furthermore, Cav1.2 dependent Ca^2+^ influx contributes to the Wnt/β-catenin activity for the ameloblastoma cell stemness and tumorigenicity. Our fundamental findings could have a major impact in the fields of oral maxillofacial surgery, and genetic manipulation or pharmacological approaches to Cav1.2 can be considered as new therapeutic options.

**Supplementary Information:**

The online version contains supplementary material available at 10.1186/s13578-022-00873-9.

## Background

Ameloblastoma (AM) is one of the most representative odontogenic epithelial tumors. Although classified as benign, the invasive growth pattern results in severe jawbone destruction, tooth loss, and metastasis to local lymph nodes or distant organs, such as the brain, lung, and skin, even though it is extremely rare [1]. Odontogenic keratocyst (OKC), a type of odontogenic lesion, were reclassified from odontogenic tumors into odontogenic cysts. Classification remains controversial due to the hallmarks of cysts and tumors both exist in OKC. Although AM and OKC are believed to originate from dental epithelial cells and share similar sites of presentation in the jawbone, they have distinct histopathological features and transcriptomic expression [2, 3].

It is no doubt that the primary factor for cancer onset might be mutations in the oncogenes or tumor suppressor genes, which are directly involved with cell division or cell death, then in any other gene potentially affected by genomic instability. However, among genes affected during oncogenic transformation, it is inevitable that ion channel coding genes promote the transition to more aggressive cancer phenotype [4]. Calcium signaling was initially described as a regulator of various cellular processes, including cell proliferation, differentiation, and gene expression [5]. However, aberrant intracellular calcium signaling is considered crucial in cancer onset and progression [6]. Accumulating evidence indicates that one or several calcium channels are modified in expression and/or activity in different cancer cells and participate in most oncogenic processes driving the malignant phenotype [7–9]. The L-type voltage-gated calcium channel (VGCC) is an integral cell membrane protein complex that selectively mediates the influx of Ca^2+^ into the cell in response to membrane depolarization [10]. It is mostly expressed in excitable tissues, such as the heart, muscle, and brain [11]. However, it has been reported that Cav1.2 (*CACNA1C*, a subunit of L-type VGCC) also participates in the progression of several cancers (breast cancer and oral squamous carcinoma) and is strongly correlated with the poor prognosis [12, 13]. Nuclear factor of activated T cells (NFAT) is a family of transcription factors in which NFATc1 is a well-known Ca^2+^-dependent transcription factor [14] that promotes cell proliferation in ovarian cancer and pancreatic ductal adenocarcinoma [15, 16]. Despite numerous studies examining calcium signaling in cancer, calcium signaling studies in AM are limited [17].

Cells that possess self-renewal capacity and differentiation potential in cancer and contribute to multiple tumor malignancies are termed cancer stem cells (CSCs) [18]. It has been well documented that calcium channels are highly associated with CSC processes such as proliferation, self-renewal, and differentiation [5], and inhibition of Cav1.2 has been shown to suppress CSC properties in non-small cell lung cancer [9]. However, no information is available regarding the role of Cav1.2, including the properties of CSCs, in AM tissues or cells.

Throughout the comparative transcriptome analysis between AM and OKC, L-type VGCCs were clearly upregulated in AM, which allowed to lead Ca^2+^ influx in primary AM cells. Furthermore, gain- and loss-of-function analysis in CACNA1C verified that Cav1.2 controlled AM cell proliferation. Evidently, the maintenance of stemness was highly associated with Cav1.2 that was confirmed by tumoroid analysis in detail. Taken together, these studies reveal insight into unraveled roles of Cav1.2 in AM progression.

## Results

### Transcriptome analysis and cellular differentiation in AM compared to OKC

**Fig. 1:**
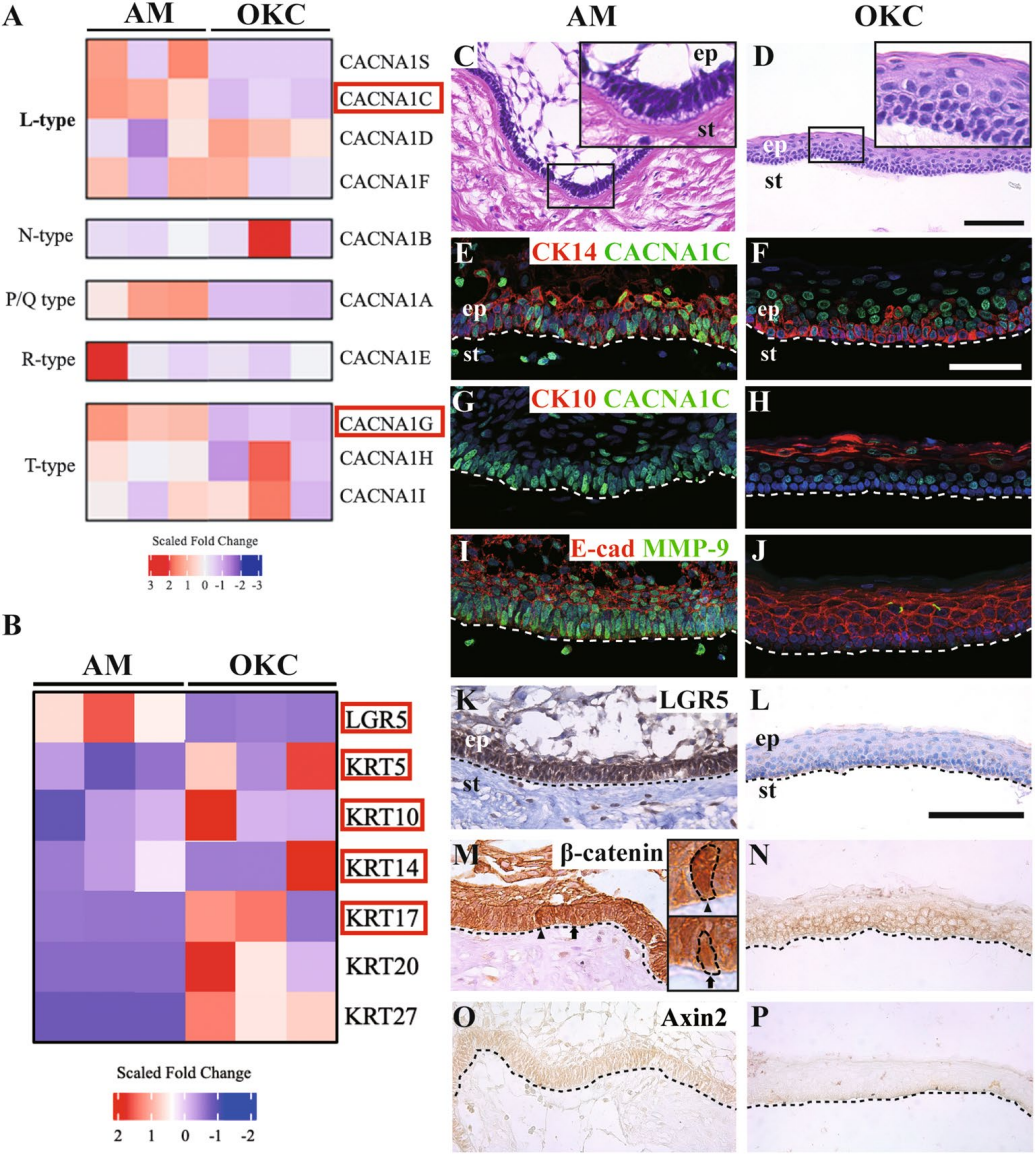
Transcriptomic and histological comparison between AM and OKC. **A**, **B** Heatmap of differentially expressed genes (DEG) between AM and OKC tissues. Significantly different genes (adj. *p* < 0.01, |Fold change| > 2) were labeled in red boxes. **A** L-type and P/Q type VGCCs were upregulated in AM, and N-type, R-type and T-type VGCCs showed a high deviation within a group. **B** LGR5 was enriched in AM, and KRT 5, 10, 14, 17, 20, and 27 were upregulated in OKC. **C**, **D** Hematoxylin and eosin staining performed on AM and OKC patient samples. AM epithelial island shows peripheral palisade cells surrounding a central area of stellate-shaped cells. The high magnification images demonstrate the nuclei displaced away from the basement membrane. The thin, regular lining of parakeratinized stratified squamous epithelium with palisading hyperchromatic basal cells (high magnification) was observed in the OKC sample. **E** In the immunofluorescence staining, CACNA1C was intensely expressed in CK14 positive peripheral cells of AM. **F** Moderate expression of CACNA1C was observed in the CK14-labeled basal layer of OKC. **G** The terminal differentiation marker CK10 is negatively expressed in the CACNA1C positive cell enriched region of AM. **H** Intensive expression of CK10 was observed in the supra-basal cell layer of OKC. **I**, **J** E-cadherin showed moderate expression in AM, and intensive expression in OKC. MMP-9 showed intensive expression in basal cell layer of AM, and negative expression in OKC. Nuclei were stained with TO-PRO-3 (TB3). **K**, **L** The stem cell marker LGR5 showed intensive expression in the peripheral cell layer of AM and showed negative expression in OKC. **M**, **N** Intense expression of β-catenin and location in nucleus (Arrow and arrowhead) were observed in peripheral layer of AM and it expressed in the adherence junctions in OKC. **O**, **P **Moderate expression of Axin2  in epithelial tumor mass of AM, and not observed in OKC. The white or black dotted lines represented the interface of the epithelium and mesenchyme tissues. Scale bar: C, D, 100 μm; E-J, 50 μm; K-P, 100 μm. *ep* epithelium, *st* stroma.

To investigate the differences in global gene expression between AM and OKC, we performed bulk RNA sequencing analyses of AM (n = 3) and OKC (n = 3) samples obtained from six individual patients. Gene ontology (GO) analysis revealed that among the upregulated genes, calcium-related GO terms were significantly enriched in AM (data not shown). The different expression pattern of VGCC-related genes between AM and OKC (Fig. 1 A; Additional file 1: Fig. S1A). Among several types of VGCCs, L-type and P/Q-type genes were upregulated in AM. The N-type, R-type, and T-type VGCCs showed individual variation within each group. The stem cell marker LGR5 was enriched in AM, while the expression of epidermal differentiation markers, including KRT10, were upregulated in OKC. Although the individual difference could be shown within a heatmap (Fig. 1B), the volcano plot displays significantly higher expression of epidermal differentiation markers in OKC (Additional file 1: Fig. S1B). Differences in histopathology and molecular expression between AM and OKC were analyzed in patient-derived samples. Epithelial islands consisting of peripheral columnar cells and stellate reticular-like cells were observed in AM (Fig. 1 C). A thin, regular lining of parakeratinized stratified squamous epithelium with palisading hyperchromatic basal cells was observed in OKC (Fig. 1D). All the patient derived AM and OKC samples showed consistent phenotype respectively (Additional file 1: Fig. S2). CACNA1C (encoding Cav1.2) was dominantly expressed in the CK14 positive peripheral epithelial cell layer in AM and was rarely observed in the CK14 positive basal cell layer in OKC (Fig. 1E, F). The terminal differentiation marker CK10 was negatively expressed in AM and intensively expressed in the suprabasal layer of OKC (Fig. 1G, H). Moderate expression of the epithelial and mesenchymal transition (EMT) marker E-cadherin and intensive expression of MMP-9 were observed in the peripheral cell layer of AM (Fig. 1I). In contrast, E-cadherin was strongly expressed in the lining epithelium, and MMP-9 was negatively expressed in OKC (Fig. 1 J). LGR5 was intensively expressed in the peripheral cell layer of AM and was not expressed in OKC (Fig. 1 K, L). In transcriptome analysis of Wnt signaling-related genes, *CTNNBIP1 (β-catenin inhibitor)* was downregulated in AM, as well as other Wnt molecules, and their transcription factors were upregulated in AM (Additional file 1: Fig. S1C). The Wnt signaling transduction molecules, such as β-catenin and Axin2, were examined between AM and OKC samples. Nuclear translocated β-catenin was detected in the peripheral layer of AM but not in OKC (Fig. 1 M, N). Moderate expression of Axin2 was observed in AM, but negative in OKC (Fig. 1O, P). The relative mRNA expression levels of *CACNA1C*, *CTNNB1*, and *LGR5* were significantly increased in AM (Additional file 1: Fig. S3). These results indicate that the expression of Cav1.2 is concentrated in the peripheral cell layer facing the connective tissue in the epithelium of AM, consistent with the location showing CSC properties such as stemness maintenance and EMT.

### Ca^2+^ influx via L-type VGCC in primary AM cells

**Fig. 2:**
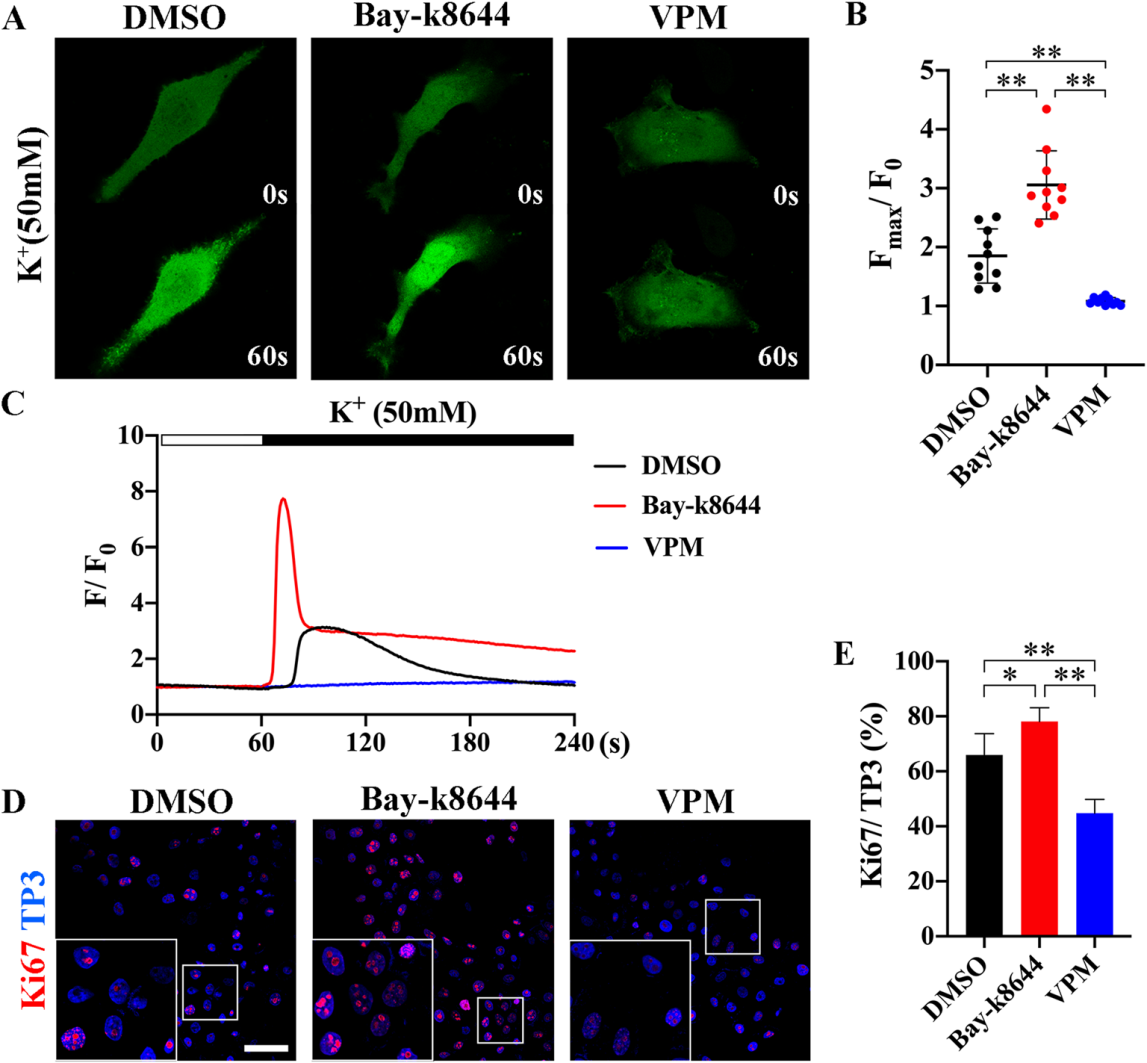
Ca^2+^ influx was mediated by L-type VGCC in AM cells. **A** Representative images of Ca^2+^ intensity in AM cells treated with the DMSO, Bay-k8644 (10 nM), or VPM (10 µM). The Ca^2+^ transients are indicated with Fluo-4 AM (green), and the depolarization was induced by K-gluconate (50 mM) solutions. Calcium imaging was obtained at 1 s intervals for a total duration of 4 min. **B** F_max_/F_0_ (F_max_: Maximum fluorescence intensity upon stimulation) ratios in the Bay-k8644 treatment group was significantly higher than those in the DMSO or VPM treatment group (N = 10 per group, biological replication). **C** Fluo-4 imaging of the Ca^2+^ response to K-gluconate in the presence of DMSO, Bay-k8644, or VPM at each time point. Note that the horizontal bar indicates the time of K-gluconate addition. The raw data are expressed as F/F_0_ (F: fluorescence intensity; F_0_: mean fluorescence intensity before stimulation). **D** Immunocytochemistry of the proliferation marker Ki67 in primary AM cells with the presence of DMSO, Bay-k8644, or VPM. White rectangle indicates the high magnification images of Ki67 positive cells. Nuclear was counterstained with TO-PRO-3 (TP3). **E** Percentage of Ki67 positive cells were quantified from immunostained images (N = 5 per group, biological replication). The Ki67 positive cell percentage in Bay-k8644 treatment group was dramatically increased compared to that in the DMSO or VPM treatment group. Scale bar: 50 μm. *p < 0.05, **p < 0.01

To identify the function of L-type VGCCs in AM progression, the depolarization of primary AM cells was confirmed with Bay-k8644 (10 nM, L-type VGCCs agonist), verapamil (VPM, 10 µM, L-type VGCCs blocker) or DMSO (negative control). The cytotoxicity of VPM was confirmed in AM cells (Additional file 1: Fig. S4). Calcium imaging was performed to evaluate the calcium influx level during depolarization of primary AM cells (Fig. 2 A). Depolarization was induced by K-gluconate solution (50 mM), and Ca^2+^ transients were indicated by Fluo-4 AM. The Ca^2+^ response to K-gluconate in the Bay-k8644 treatment group was significantly active and lasted for longer than that in the DMSO treatment group (Fig. 2 C). In contrast, no fluorescence was detected in the VPM treatment group at each time point after depolarization. The F_max_/F_0_ values in the Bay-k8644 group was 2-fold higher in the DMSO group and 7-fold higher than those in the VPM treatment group (Fig. 2B). In addition, differential proliferative properties of primary AM cells were observed in the presence of DMSO, Bay-k8644, and VPM (Fig. 2D). The number of Ki67 positive proliferating cells was dramatically increased in Bay-k8644 cells and decreased in VPM compared to that in DMSO (Fig. 2E). These results indicate that the Ca^2+^ influx in primary AM cells was mediated by L-type VGCCs, which enhanced the cell proliferation.

### Cav1.2-dependent Ca^2+^/NFATc1 signaling coupled with cell proliferation

**Fig. 3:**
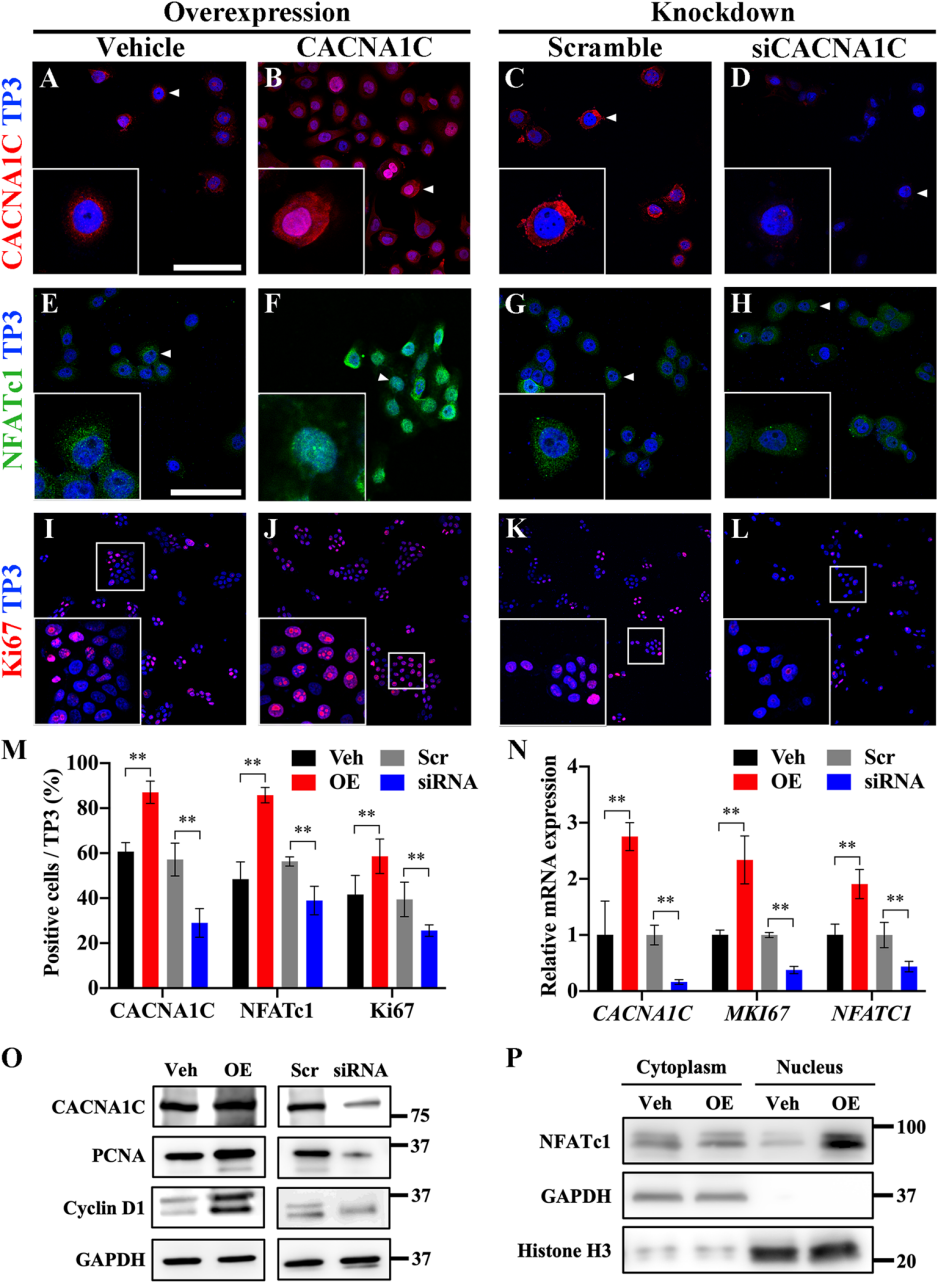
Cav1.2-dependent Ca^2+^/NFATc1 signaling increased the proliferation of AM cells. **A**–**D** Representative CACNA1C expression in AM cells indicated by arrowhead. CACNA1C was intensely expressed in the plasma membrane in the vehicle group. And it was broadly expressed in the plasma membrane, cytoplasm and nucleus in overexpression group. CACNA1C negatively expressed in knockdown group compared to the scramble.  **E**–**H** The nuclear translocation of NFATc1 was observed in the overexpression group compared to the vehicle. NFATc1 was negatively expressed in the knockdown group compared to the scramble. **I**–**L** Representative confocal images of Ki67 staining of AM cells among the vehicle, overexpression, scramble, and siRNA groups. Nuclear were stained with TO-PRO-3 (TP3). **M** Percentages of CACNA1C^+^ and NFATc1^+^ cells were quantified from immunostained images (N = 5 per group, biological replication). **N** Relative mRNA expression of *CACNA1C*, *MKI67*, and *NFATC1* between vehicle and CACNA1C overexpression, or scramble and siRNA group. **O** Western blot assay of AM cells with CACNA1C, PCNA, Cyclin D1, and GAPDH antibodies. **P** Western blot assay of nucleus and cytoplasm fractionated AM cells with NFATc1, Histone H3, and GAPDH antibodies. Scale bar: A–D, 50 μm; E–L, 100 μm. Quantitative data are presented as the mean ± SD. **p < 0.01. *Veh* vehicle, *OE* CACNA1C overexpression, *Scr* Scramble, *PCNA* Proliferating cell nuclear antigen, *NFATc1* Nuclear factor of activated T cells 1

Several studies have reported that Ca^2+^ signaling is involved in cancer cell proliferation [19–21]. To clarify the role of Cav1.2 in AM cells, we overexpressed or knocked down CACNA1C in AM cells in vitro. The Ca^2+^ influx was increased in CACNA1C-overexpressed AM cells (Additional file 1: Fig. S5). CACNA1C was broadly expressed in the cell membrane and nucleus in the CACNA1C overexpression group compared to that in the vehicle group (Fig. 3 A, B). The expression of CACNA1C in the knockdown group was significantly lower than that in the scramble group (Fig. 3 C, D). As a Ca^2+^-dependent transcription factor, NFATc1 was primarily found in the cytoplasm. The nuclear accumulation of Cav1.2 detected in CACNA1C-overexpressed AM cells indicates that NFATc1 was activated by Cav1.2 (Fig. 3E, F). NFATc1 expression was dramatically decreased by the inhibition of CACNA1C compared to the scrambled control (Fig. 3G, H). The proliferation marker Ki67 was significantly increased in the overexpression group and decreased in the knockdown group compared to that in the vehicle and scramble groups, respectively (Fig. 3I–L). The percentage of positive cells indicated a correlation between CACNA1C and NFATc1 expression (Fig. 3 M). Furthermore, the mRNA expression levels of CACNA1C, MKI67, and NFATc1 were up-regulated in the overexpression group and down-regulated in knockdown group (Fig. 3 N). In addition, the protein expression of CACNA1C, PCNA, and cyclin D1 was increased in the CACNA1C overexpression group and decreased in the knockdown group compared to the vehicle and scramble groups, respectively (Fig. 3O). The expression level of cytoplasmic NFATc1 was not significantly different between the vehicle and overexpression groups; however, nuclear translocation of NFATc1 was dramatically increased in the overexpression group (Fig. 3P). The cell proliferation was also confirmed by PCNA expression in the 3-dimensionaly-cultured AM tumoroids (Additional file 1: Fig. S6A), The PCNA positive cell were dramatically increased in CACNA1C-overexpressed AM tumoroids and significantly decreased in the VPM treatment group (Additional file 1: Fig. S6B). We also confirmed the expression of NFATc1 was up-regulated in AM compared to the OKC, which is consisted with in vitro results (Additional file 1: Fig. S6C). Altogether, Cav1.2-dependent Ca^2+^/NFATc1 signaling promotes the proliferation of AM cells.

### Function of Cav1.2 in maintenance of Wnt/β-catenin activity

**Fig. 4:**
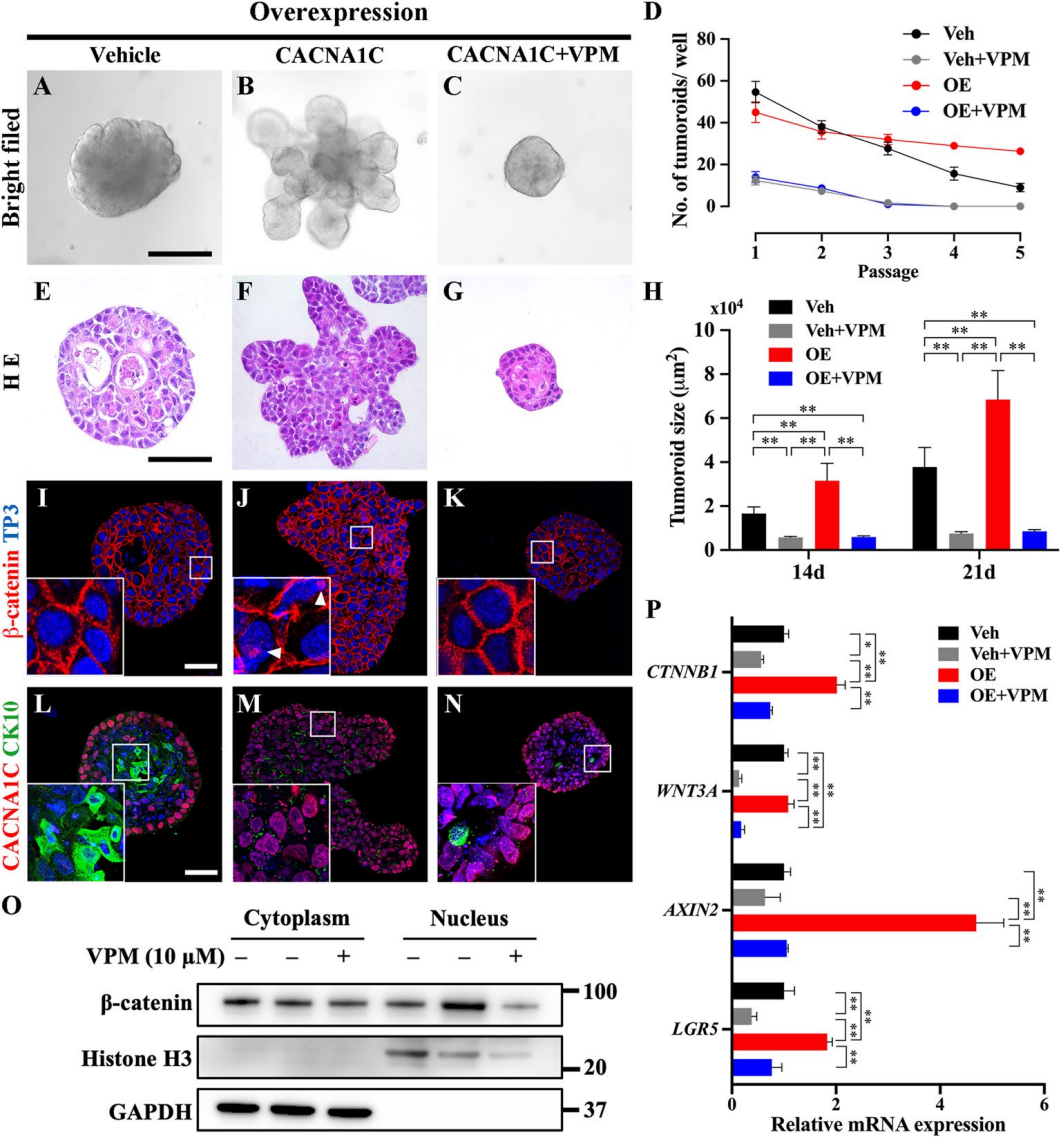
Cav1.2 maintained Wnt/β-catenin signaling activity in AM tumoroids. **A**–**C** Bright field images of an AM tumoroid generated from a single cell. **D** The organoid forming efficiency was evaluated as a percentage of the number of tumoroids in each well. The CACNA1C overexpression effectively retained the organoid forming efficiency compared to vehicle and VPM treatment group. **E**–**G** Hematoxylin and eosin staining of paraffin sections of AM tumoroids. Spherical shaped AM tumoroids were observed in vehicle and VPM treatment group. Budding-like structures expended from CACNA1C-overexpressed AM tumoroids. **H** Quantification of the size of 14 days and 21 days cultured AM tumoroids. **I**–**K** Immunohistochemistry staining of β-catenin in an AM tumoroid. β-catenin was primary found in the plasma membrane. The nuclear accumulation of β-catenin was dominantly observed in the overexpression group compared to the vehicle and VPM treatment groups. **L**–**M** Immunohistochemistry staining of CK10 and CACNA1C. The white rectangle indicates the CK10 positive differentiated AM cells in the vehicle and VPM treatment groups. Nuclei were counterstained by TO-PRO-3 (TP3). **O** Western blot assay of nucleus and cytoplasm fractionated AM tumoroids with β-catenin antibody. **P** The relative mRNA expression of *WNT3A, AXIN2, CTNNB1*, and *LGR5* in CACNA1C-overexpressed and VPM treated tumoroids. Scale bar: A–C, 300 μm; E–G, I–K, L–M, 100 μm. Data are presented as the mean ± SD of triplicate experiments. *p < 0.05; **p < 0.01. Veh: Vehicle, OE: CACNA1C overexpression

Three-dimensional (3D)-culture can recapitulate physiologically relevant phenotypes of the tissue of origin [22]. Recent years have seen great advancements in establishing different organoid or tumoroid models from various organs and cancers, including AM tumoroids [23, 24]. CACNA1C overexpressing AM cells 3-dimensionaly were cultured with the presence of L-type VGCC blocker, VPM. Spherical AM tumoroids were observed in the vehicle group (Fig. 4 A), and several budding-like structures extended from the CACNA1C-overexpressed AM tumoroid (Fig. 4B). The budding-like structure was disappeared in the vehicle and CACNA1C-overexpressed AM tumoroid with the presence of VPM (Fig. 4 C; Additional file 1: Fig. S7A). To evaluate the effect of Cav1.2 on AM cell tumorigenicity, the organoid-forming efficiency was calculated as the number of tumoroids per well (Fig. 4D). With an increase in the number of passages, the organoid formation efficiency in all groups decreased to varying degrees. The vehicle + VPM group and the overexpression + VPM group showed low organoid forming efficiency from the passage 1. The overexpression of CACNA1C effectively retained the organoid-forming efficiency compared to the vehicle or VPM treatment groups. A keratinizing core was observed in the round-shaped tumoroids (Fig. 4E). Budding-like structures were generated from the tumoroid (Fig. 4 F) but were dismissed by the presence of VPM (Fig. 4G; Additional file 1: Fig. S7B). The size of the tumoroid in the overexpression group was significantly increased in both the 14- and 21-day cultures compared to the vehicle and VPM treatment groups (Fig. 4 H). Interestingly, the overall tumoroid sizes were very similar between the vehicle + VPM group and the overexpression + VPM group. Furthermore, dominant expression of nuclear translocated β-catenin indicated high Wnt/β-catenin signaling activity in the overexpression group compared to vehicle and VPM treatment groups (Fig. 4I–K; Additional file 1: Fig. S7C). Previous study reported that pharmacologically or genetically inhibits the T-type calcium channel Cav3.2 in glioblastoma result in a reduce of CSC population via promotes the CSC differentiation [25]. Similarly, keratinization was predominantly observed in the suprabasal layer, as indicated by the intense expression of CK10 (Fig. 4 L). However, CK10 was negatively expressed in CACNA1C-overexpressed AM tumoroids, indicating that AM cell differentiation was suppressed by increased Cav1.2 (Fig. 4 M). Both the vehicle + VPM group and overexpression + VPM group showed faint CK10 expression (Fig. 4 N; Additional file 1: Fig. S7D). Next, a western blot assay was performed to confirm the effect of Cav1.2 on the maintenance of Wnt/β-catenin signaling activity. The nuclear accumulation of β-catenin in the overexpression group was dramatically increased compared to that in the vehicle and VPM groups (Fig. 4O). Furthermore, the mRNA expression of canonical Wnt signaling molecules, including *CTNNB1*, *AXIN2* and the Wnt signaling enhancer *LGR5*, was upregulated by CACNA1C overexpression and downregulated by VPM treatment (Fig. 4P). The expression of *WNT3A* was not affected by CACNA1C overexpression, but dramatically downregulated by VPM treatment in both the Veh + VPM and OE + VPM groups. Thus, it appears that the expression of Cav1.2 plays an essential role in retaining Wnt/β-catenin signaling activity in AM cells, and represents a crucial element for AM cell stemness.

**Fig. 5:**
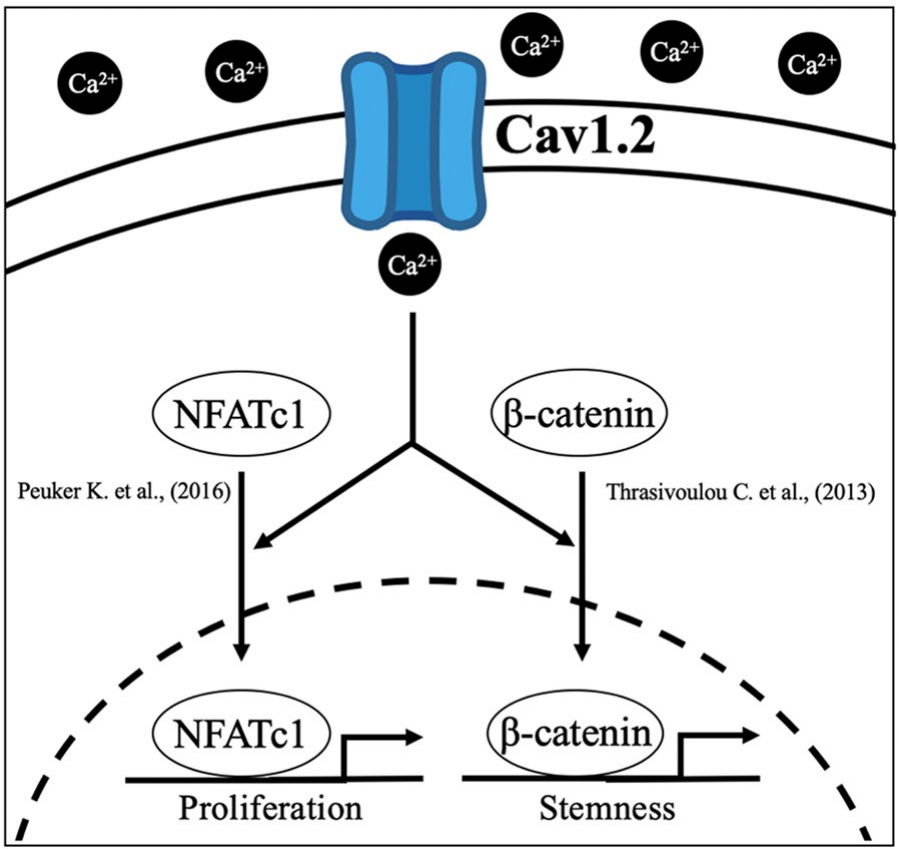
Schematic summary. Cav1.2 is dominantly expressed in ameloblastoma cells compared to the OKC. The Cav1.2-dependent Ca^2+^ influx increases the proliferation of ameloblastoma cells by the accumulation of nucleic NFATc1, while intracellular Ca^2+^ enhances Wnt/β-catenin signaling activity and the maintenance of the stemness of the ameloblastoma cells

## Discussion

In the present study, comparative transcriptomic profiling of two representative odontogenic lesions, AM and OKC was performed for the first time. Since AM is considered to originate from dental epithelial cells, compared with the different-origin surrounding tissue may mislead the transcriptomic analysis result. A previous study reported that normal adjacent tissue to the tumor has unique characteristics differentiating it from healthy tissues [26]. Thus, OKC is an ideal control for the transcriptomic comparison with AM owing to the same origin and different classification. Remarkably, the L-type and P/Q type VGCCs were significantly upregulated in AM compared to OKC. However, a variety of cancers, including breast cancer, are associated with the modulation of L-type VGCCs [21], and the AM stem/progenitor cell marker LGR5 was significantly upregulated in AM, which is similar to the results of a previous study [23]. The results of immunohistochemical analysis of AM and OKC tissues, including CK10, CK14, E-cadherin, MMP-9, and LGR5, are consistent with those of previous studies [23, 27, 28]. However, we found that CACNA1C was co-expressed with the general progenitor marker CK14 and was negatively correlated with the terminal differentiation marker CK10 in AM. Furthermore, CACNA1C positively correlated with MMP-9 and LGR5 expression. Owing to the importance of milk calcium in neonatal development, the breast is intrinsically related to Ca^2+^. Indeed, various calcium channels involved in transporting Ca^2+^ into milk are altered or modulated and may even be specific to breast cancer molecular subtypes. Similarly, dental epithelial cells, which are thought to be the origin of AM, possess many calcium channels required for tooth development [29].

Our results imply an association between Cav1.2 and the characteristics of AM stem/progenitor cells. A recent research has shown that alteration in intracellular Ca^2+^ is important in the self-renewal capacity, proliferation and differentiation of stem cells, with implication of various Ca^2+^ channels including VGCC [9, 30]. We demonstrated that the Ca^2+^ influx increased in the presence of an L-type VGCC agonist (Bay-k8644) and decreased by the blocker (VPM) in primary AM cells in vitro. Furthermore, we observed that AM cell proliferation was positively correlated with both L-type VGCC activity and the expression of Cav1.2 in the gain-of-function or loss-of-function study. NFAT is a family of transcription factors in which inactivated NFATc1 can be dephosphorylated by Ca^2+^/calcineurin signaling and translocated to the nucleus, leading to transcriptional activation [31]. It is known that the activity of NFAT is regulated by L-type VGCCs in neuronal cells [32]. Furthermore, the proliferation and anchorage-independent growth of pancreatic tumor cells are dependent on calcineurin activity and high levels of nuclear NFATc1 [33]. In colorectal cancer progression, calcineurin supported the survival and proliferation of CSCs in a NFAT-dependent manner [34]. We also observed a significant upregulation of NFATc1 in AM compared to that in OKC through the transcriptome analysis results (Additional file 1: Fig. S6C). Remarkably, we found that CACNA1C showed a positive correlation with NFATc1 expression, as well as an increase in cell proliferation. We also examined the nuclear translocation of NFATc1 in the CACNA1C-overexpressed AM cells. Indeed, AM is clinically characterized as a slow growing tumor, even though the present study showed the higher expression of Ki67 and Cyclin D1 was shown in the present study. Without medical treatment, AM shows an unlimited growth tendency in general. Numerous reports about the upregulation of L-type VGCC in several cancers [12, 13] including our results clearly indicate that Cav1.2-mediated  Ca^2+^ influx promotes the nuclear translocation of NFATc1, that is followed by leading to an increase of AM cell proliferation.

Several studies have shown that the interplay between calcium channels and Wnt signaling is critical for the maintenance of stem cells. Impairment in the activity of TRPV channels is related to decreased Ca^2+^ flux in bone marrow mesenchymal stem cells, which ultimately downregulates Wnt/β-catenin signaling, leading to the dysregulation of osteogenic differentiation [35]. Downregulation of Cav1.2 has been shown to induce age-related osteoporosis by suppressing the canonical Wnt signaling pathway [36]. Ca^2+^ dynamics can finely modulate β-catenin nuclear translocation [37]. Intracellular Ca^2+^ enables β-catenin to pass through the nuclear membrane by neutralizing negatively charged β-catenin and activating Wnt pathway target genes [38]. We have previously shown that activation of Wnt signaling results in the hyper-differentiation of CSCs in AM-1 cells [24]. The organoid-forming assay showed strong tumorigenicity in Cav1.2-overexpressed AM tumoroids. It indicates that Cav1.2 overexpression retains the self-renewal of AM stem/ progenitor cells. A similar result had been reported that knockdown of VGCC in hepatocellular carcinoma reduced the self-renewal and tumor formation capacities of CSCs [39]. The nuclear accumulation of β-catenin and the upregulation of Wnt signaling molecules in CACNA1C-overexpressed AM tumoroids implies that Cav1.2-dependent intracellular Ca^2+^ plays an essential role in retaining Wnt/β-catenin signaling activity. In VPM treated groups, the tumoroid forming efficiencies, average sizes of tumoroids and even Wnt/β-catenin signaling activities were almost the same regardless of overexpression of Cav1.2. In addition, both the VPM treatment groups have a higher degree of differentiation (CK10) than the overexpression group, but appear to be significantly lower than that of the negative control (vehicle). It indicates that the VPM groups cannot progress to the differentiation stage because tumor progression is suppressed in the tumoroid formation stage. To sum it up, Cav1.2-dependent Ca^2+^ signaling contributes to the maintenance of AM cell stemness, on the contrary the VPM leads to retardation in whole process of tumoroid formation effectively.

A few studies have been suggested that tumorigenesis is closely associated with mutations in Ca^2+^ permeable channels such as Orai1/Stim1 or Na+/Ca2 + exchanger (NCX) [40, 41]. However, a direct connection between VGCC mutation and the initiation of AM has not been reported. Also, the relationship between calcium signaling and initiation of AM was not revealed in the present study. Nevertheless, gain-of-function mutation of VGCC is more related to tumor maintenance and proliferation than tumorigenesis.

In conclusion, we suggest that Cav1.2 regulates the NFATc1 nuclear translocation to enhance AM cell proliferation. Furthermore, Cav1.2 dependent Ca^2+^ influx contributes to the Wnt/β-catenin activity for the AM cell stemness and tumorigenicity (Fig.5). However, a clear explanation that how Cav1.2 dependent Ca^2+^ signaling decides to promote cell proliferation or enhance the Wnt/β-catenin activity was not provided. Further study is needed to determine whether the presented mechanism governs in other bone invasive cancers. Then, genetic manipulation or pharmacological approaches to Cav1.2 can be considered as new therapeutic options.

## Methods

### Tissue collection

This study was approved by the institutional review board (IRB) at the University of Yonsei (2-2018-0050) and followed by human subject research guidelines and a protocol. Fresh three AM and three OKC samples were obtained from six individual patients during post-surgical procedures following appropriate informed consent who underwent treatment at the Department of Oral and Maxillofacial Surgery, Yonsei University Dental Hospital. Diagnoses were made by two independent pathologists, including a board-certified oral and maxillofacial pathologist.

### RNA sequencing

Total RNA was extracted from AM and OKC tissues using TRIzol® Reagent (#15596-026, Thermo Fisher Scientific, USA) respectively. The RNA was stored at -70 °C and measured at an optical density of 260 nm. The mixtures of total RNA were incubated with Oligo dT (Gibco BRL, Rockville, NY, USA). The library was constructed and sequenced using an Illumina HiSeq2500 sequencer (Illumina, CA, USA). Differentially expressed genes (DEGs) between the AM and OKC were identified using the R package for RNA-seq data analysis, DESeq2 [42]. Based on significant DEGs (adjusted p-value < 0.01), gene ontology (GO) analysis was performed using a R package for comparison of biological themes in gene clusters, clusterProfiler [43]. The steps were followed as previously described [44].

### Cell cultures

The primary AM or OKC cells were isolated as previously described [23]. The suspended primary AM cell were seeded in the 0.1% gelatin-coated (PRIMARY CELL SOLUTION, PCS-999-027) culture dishes (1×10^6^) in defined Keratinocyte Basal Medium-2 (KBM-2, LONZA, CC-3103) supplemented with KGM^Ⓡ^-2 SingleQuots^Ⓡ^ (LONZA, CC-4152) at 37 °C in a humidified atmosphere with 5% carbon dioxide (CO_2_). After 48 h, the non-adherent cells were removed, and fresh KGM^Ⓡ^-2 media were replenished every 3 days. Early passages of primary cells were cryopreserved, and less than six passages were used for further experiments. For calcium imaging experiment or histological analysis, AM cells were seeded in the KGM^Ⓡ^-2 media with DMSO (0.1% v/v), Bay-k8644 (10 nM) or VPM (10 µM) and cultured for 12 h.

### Overexpression of CACNA1C in AM cells

The full-length protein-coding human CACNA1C cDNA (Horizon Discovery, MHS6278-202857128) was cloned in pCDH-CMV-MCS-EF1-CopGFP-T2A-Puro Lenti viral vector. The pLenti-Puro expression vector, psPAX2 packaging vector and pMD2.G envelope vector were co-transfected into HEK-293 cells using Lipofectamine LTX. Then, the transfection medium was removed, and HEK-293 cells were cultured in RPMI 1640 + 10% FBS for 48 h. Virus particles were syringe-filtered through a 0.45-µm-nylon filter, and Polybrene (Santa Cruz) was added to a final concentration of 8 µg/mL. AM cells were cultured in lentiviral media and left to allow infection for 48 h. Transduced cells were harvested after Puromycin (1.5 µg/mL) selection for 72 h.

### Knockdown of CACNA1C in AM cells

AM cells were prepared on 0.1% gelatin-coated 6 well plates at a density of 200,000 cells/well in KGM-2. *CACNA1C* siRNA (sc-42688, Santa Cruz) or Control siRNA (scrambled, sc-37007 Santa Cruz) was added to Opti-MEM (Gibco) at 20 nM concentration. RNAiMAX (Invitrogen) was also added to the Opti-MEM by 0.125 µL per unit area (cm^2^) of culture dish according to the manufacturer’s instructions. AM cells were incubated with siRNA containing Opti-MEM for 6 h. The cells were gently washed with DPBS then the medium was changed to fresh KGM^®^-2. Cells were harvested for further analyses at 72 h after knockdown.

### Calcium imaging

Calcium imaging was performed as previously described with slight modifications [45]. In brief, AM cells were seeded in 35-mm confocal dishes (SPL Life Sciences) at a density of 3 × 10^6^ cells, and the non-adherent cells were removed after 12 h. The AM cells were washed with HBSS buffer containing Ca^2+^ and Mg^2+^ ions (Gibco), loaded with 5 µM Fluo-4, AM (Invitrogen) in HBSS buffer containing 0.01% Pluronic F-127 (Invitrogen), and incubated at 37 °C for 30 min. The calcium influx and resting Ca^2+^ levels were measured in the HBSS buffer. Before imaging, cells were washed four times with HBSS buffer. The fluorescence of Fluo-4 was excited at wavelengths of 494 nm every 1 s by means of a high-speed wavelength device. Images were recorded using a Leica DMi8 confocal microscope. To minimize bleaching, the intensity of the excitation light and sampling frequency were kept as low as possible, and 30 cells were analyzed for each experimental condition.

### Tumoroid formation

Single cell suspended AM cells (vehicle transduction) or CACNA1C overexpressed AM cells were directly dispersed into Matrigel (Corning Life Sciences) at a density of 2500 cells per 25 µL Matrigel drop. The dish was inverted during the solidification of the Matrigel to prevent the cells from attaching to the culture dish. After solidification for 15 min, the mixture of cells and Matrigel was cultured in an AM-tumoroid culture medium containing 50% KGM^®^-2 and 50% Wnt3a-conditioned medium (obtained from WNT reporter cell line, ENZ-61002). Tumoroid formation was observed under a microscope every 2–3 days, and whole Matrigel containing tumoroids was harvested on day 21. For VPM treated groups, tumoroids were cultured in the media with VPM (10 µM) for 21 days until tumoroids being harvested.

### Organoid-forming efficiency assay

The organoid-forming efficiency was determined by quantification of the number and size of AM tumoroids. The total number of AM tumoroids per well was manually counted 21 days after seeding using a phase-contrast microscope at 4× magnification. Eight-bit binary images of whole tumoroid drops were analyzed with ImageJ (NIH) using the “analyze particles” option set to the following parameters: size, 25-infinite and circularity, 0.5–1.0 (n = 3).

### Histology

Fresh patient samples (AM and OKC) or 21-day cultured-tumoroids were immersed in 4% paraformaldehyde (PFA) for fixation. Paraffin-embedded specimens were sectioned into 5-µm thick sections. Hematoxylin and eosin (HE) staining was performed after deparaffinization. For immunological staining of histological sections or cells, the following antibodies were used, as previously described [46]. Rabbit anti-CACNA1C (1:200, Alomone Labs, ACC-003), mouse anti-CK14 (1:500, Abcam, ab7800), mouse anti-CK10 (1:500, Invitrogen, MA5-13705), mouse anti-E-cadherin (1:500, BD Biosciences, AF748), rabbit anti-MMP-9 (1:200, Merck, AB19016), rabbit anti-LGR5 (1:200, Abcam, ab75732), mouse anti-Ki67 (1:200, Abcam, ab16667), mouse anti-PCNA (1:500, Abcam, ab29), mouse anti-NFATc1 (1:200, Santa Cruz, SC-7294), and mouse anti-β-catenin (1:500, Santa Cruz, SC-7963). Target retrieval solution (DAKO, S2369) was used for antigen retrieval prior to blocking. TO-PRO-3™ Iodide (1:1000; Invitrogen, T3605) was used for counterstaining. Images were acquired using a confocal microscope (Leica DMi8, Wetzlar, Germany). Quantification analyses of Ki67, CACNA1C and NFATc1 positive cells were performed with five images (294 × 294 μm) acquired at different culturing wells, respectively (biological replication).

### Nucleus/cytoplasm fractionation and Western blot

Nucleus and cytoplasm fractions of AM cells or AM tumoroids were prepared using the NE-PER Nuclear and Cytoplasmic Extraction reagents (Thermo Scientific), following the manufacturer’s protocols. The whole-cell-lysates or fractionated proteins were separated by SDS-PAGE, transferred to PVDF membranes, blocked with 5% (w/v) skim milk in TBST (10 mM Tris, pH 7.4, 150 mM NaCl, 0.1% Tween 20) for 1 h, and probed with the Rabbit anti-CACNA1C (1:1000, Alomone Labs, ACC-003), mouse anti-PCNA (1:1000, Abcam, ab29), cyclin D1 (1:1000, Santa Cruz, SC-8396), NFATc1 (1:1000, Santa Cruz, SC-7294), β –catenin (1:1000, Santa Cruz, SC-7963), histone H3 (1:1000, Cell Signaling Technology, 3638), GAPDH (1:3000, Santa Cruz, SC-32,233) with gentle shaking at 4 °C overnight. The membranes were washed three times for 10 min each and incubated with the appropriate peroxidase-conjugated secondary antibodies (1:3000, Santa Cruz) for 1 h at room temperature. The signals were detected using the ECL system (RPN2232, GE Healthcare Life Sciences, USA), according to the manufacturer’s protocol.

### Real-time quantitative polymerase chain reaction

The total RNA was extracted using TRIzol® reagent (#15596-026, Thermo Fisher Scientific, USA). The extracts were reverse transcribed using Maxime RT PreMix (#25081, iNtRON, Korea). RT-qPCR was performed using a StepOnePlus Real-Time PCR System (Applied BioSystems, USA). The amplification program consisted of 40 cycles of denaturation at 95 °C for 15 s and annealing at 61 °C for 30 s. The expression levels of each gene are expressed as normalized ratios against the *B2M* housekeeping gene. The oligonucleotide RT-qPCR primers for *CACNA1C, LGR5, MKI67, NFATC1, CTNNB1, AXIN2, WNT3A, B2M* are as follows:

***CACNA1C***-F: 5’-GCT TAT GGG GCT TTC TTG CAC-3’; R: 5’-ACT GGA CTG GAT GCC AAA GG-3’, ***LGR5***-F: 5’-TAT GCC TTT GGA AAC CTC TC-3’; R: 5’-CAC CAT TCA GAG TCA GTG TT-3’, ***MKI67***-F: 5’-ACG CCT GGT TAC TAT CAA AAG G-3’; R: 5’- CAG ACC CAT TTA CTT GTG TTG GA-3’, ***NFATC1***-F: 5’-CAC CGC ATC ACA GGG AAG AC-3’; R: 5’-GCA CAG TCA ATG ACG GCT C-3’, ***CTNNB1***-F: 5’-CGC ACC ATG CAG AAT ACG AA-3’; R: 5’-ATC CAC TGG TGA CCC AAG CA-3’, ***AXIN2***-F: 5’-CCA AGC AGA CGA CGA AGC AT-3’; R: 5’-GTT TCC GGA GCC TTG GAG TG-3’, ***WNT3A***-F: 5’-CTA CCA GGG AGT CGG CCT TT-3’; R: 5’-AAC TCC CGA GAC ACC ATC CC-3’, ***B2M***-F:5’- GCC GTG TGA ACC ATG TGA CT-3’; R: 5’-GCT TAC ATG TCT CGA TCC CAC TT-3’.

### Statistical analysis

All statistically analyzed data were based on at least three separate experiments with consistent results. *p*-values were obtained using a 2-tailed, unpaired t-test, Tukey’s multiple comparisons test or one-way ANOVA (GraphPad Prism 8, GraphPad Software, San Diego, CA, USA). *p<*0.05 was considered statistically significant.

## Supplementary Information

**Additional file 1: Fig. S1**. Transcriptomic comparison between ameloblastomas (AMs) and odontogenic keratocysts (OKCs).** Fig. S2**. Histopathological validation for the AM and OKC samples.** Fig. S3**. Validation for the mRNA expression of selected parameters from RNA-sequencing.** Fig. S4**. Cytotoxicity of verapamil.** Fig. S5**. Ca2+ influx increased in CACNA1C-overexpressed AM cells.** Fig. S6**. The cell proliferation strongly associated with the Cav1.2 in AM tumoroid.** Fig. S7**. Cav1.2 maintained Wnt/β-catenin signaling activity in AM tumoroid.

## Data Availability

The datasets used and/or analyzed during the current study are available from the corresponding author on reasonable request. The RNA sequencing data have been deposited in the Gene Express Omnibus (GEO) database [GEO: GSE186489].

## Abbreviations

AM: Ameloblastoma
CSCs: Cancer stem cells
DPBS: Dulbecco’s Phosphate buffered saline
EMT: Epithelial-mesenchymal transition
GO: Gene ontology
NFAT: Nuclear factor of activated T cells
OKC: Odontogenic keratocyst
VGCC: L-type voltage-gated calcium channel
VPM: Verapamil
