# Supplementary material for: Unraveled roles of Cav1.2 in proliferation and stemness of ameloblastoma
Source: Cell Biosci. 2022 Sep 3;12:145. doi: 10.1186/s13578-022-00873-9 (PMC9440535; doi:10.1186/s13578-022-00873-9)
Supplement: Supplementary file 1 — Additional file 1: Fig. S1. Transcriptomic comparison between ameloblastomas (AMs) and odontogenic keratocysts (OKCs). Fig. S2. Histopathological validation for the AM and OKC samples. Fig. S3. Validation for the mRNA expression of selected parameters from RNA-sequencing. Fig. S4. Cytotoxicity of verapamil. Fig. S5. Ca2+ influx increased in CACNA1C-overexpressed AM cells. Fig. S6. The cell proliferation strongly associated with the Cav1.2 in AM tumoroid. Fig. S7. Cav1.2 maintained Wnt/β-catenin signaling activity in AM tumoroid. [file 13578_2022_873_MOESM1_ESM.docx]

**Unraveled roles of Cav1.2 in proliferation and stemness of ameloblastoma**

Shujin Li, Dong-Joon Lee, Hyun-Yi Kim, Jun-Young Kim, Young-Soo Jung, Han-Sung Jung^*^

^*^ Correspondence

Han-Sung Jung

Han-Sung Jung, Department of Oral Biology, Yonsei University College of Dentistry, 50-1 Yonsei-ro, Seodaemun-gu, Seoul 03722, South Korea.

Email: hsjung@yuhs.ac

**Contents of Additional file:**

Fig. S1 Transcriptomic comparison between ameloblastomas (AMs) and odontogenic keratocysts (OKCs).

Fig. S2 Histopathological validation for the AM and OKC samples.

Fig. S3 Validation for the mRNA expression of selected parameters from RNA-sequencing.

Fig. S4 Cytotoxicity of verapamil.

Fig. S5 Ca^2+^ influx increased in CACNA1C-overexpressed AM cells.

Fig. S6 The cell proliferation strongly associated with the Cav1.2 in AM tumoroid.

Fig. S7 Cav1.2 maintained Wnt/ β-catenin signaling activity in AM tumoroid.

**
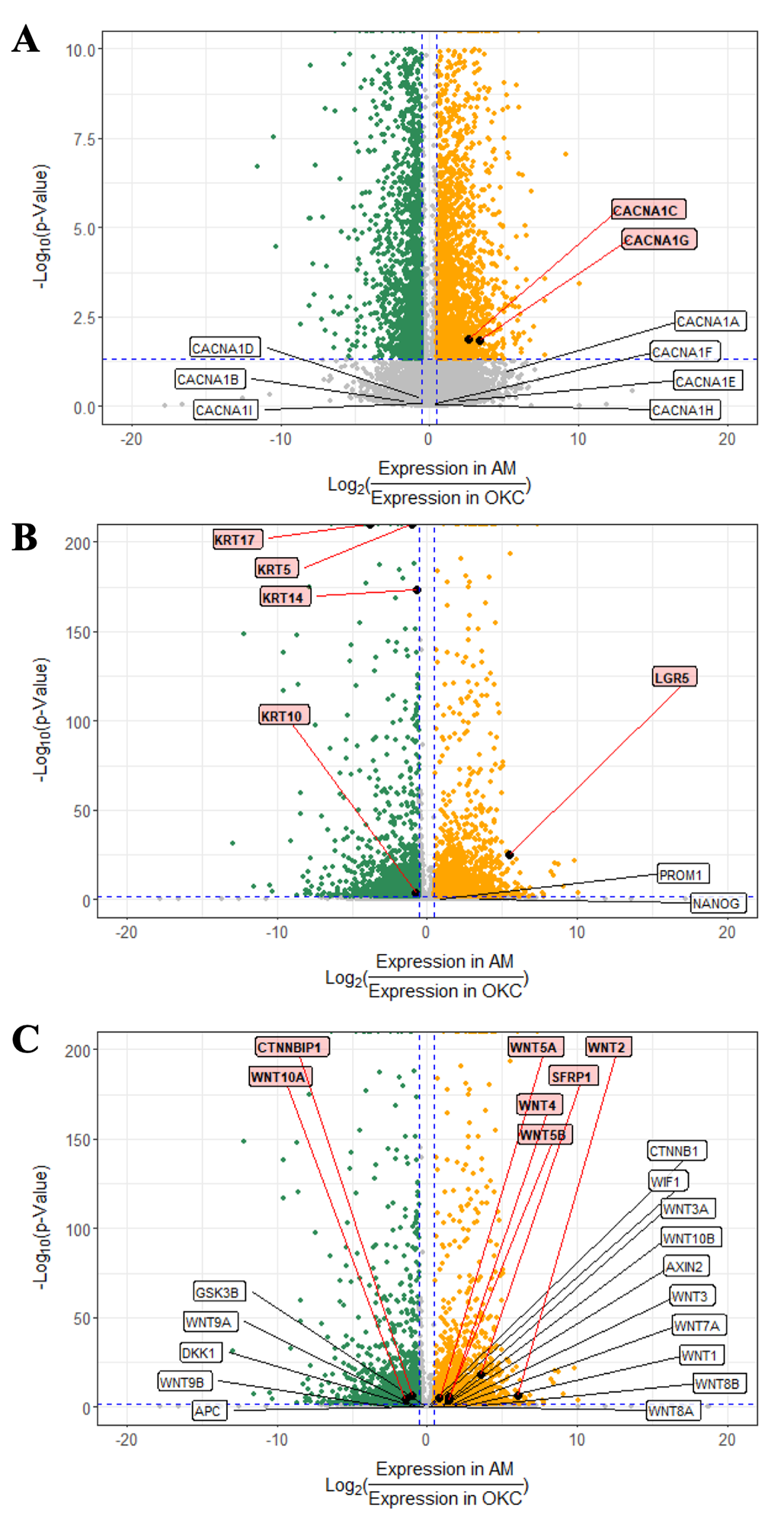
**

**Fig. S1 | Transcriptomic comparison between ameloblastomas (AMs) and odontogenic keratocysts (OKCs).**

Differentially expressed genes (DEGs) of voltage-gated calcium channels (A), differentiation and stemness (B) and Wnt signaling related genes (C) are visualized using volcano plot. A horizonal dashed line indicate *p*-value = 0.01 and vertical dashed lines mark fold change = -2 and 2, respectively. Significant genes were indicated by black dots and annotated in pink boxes.


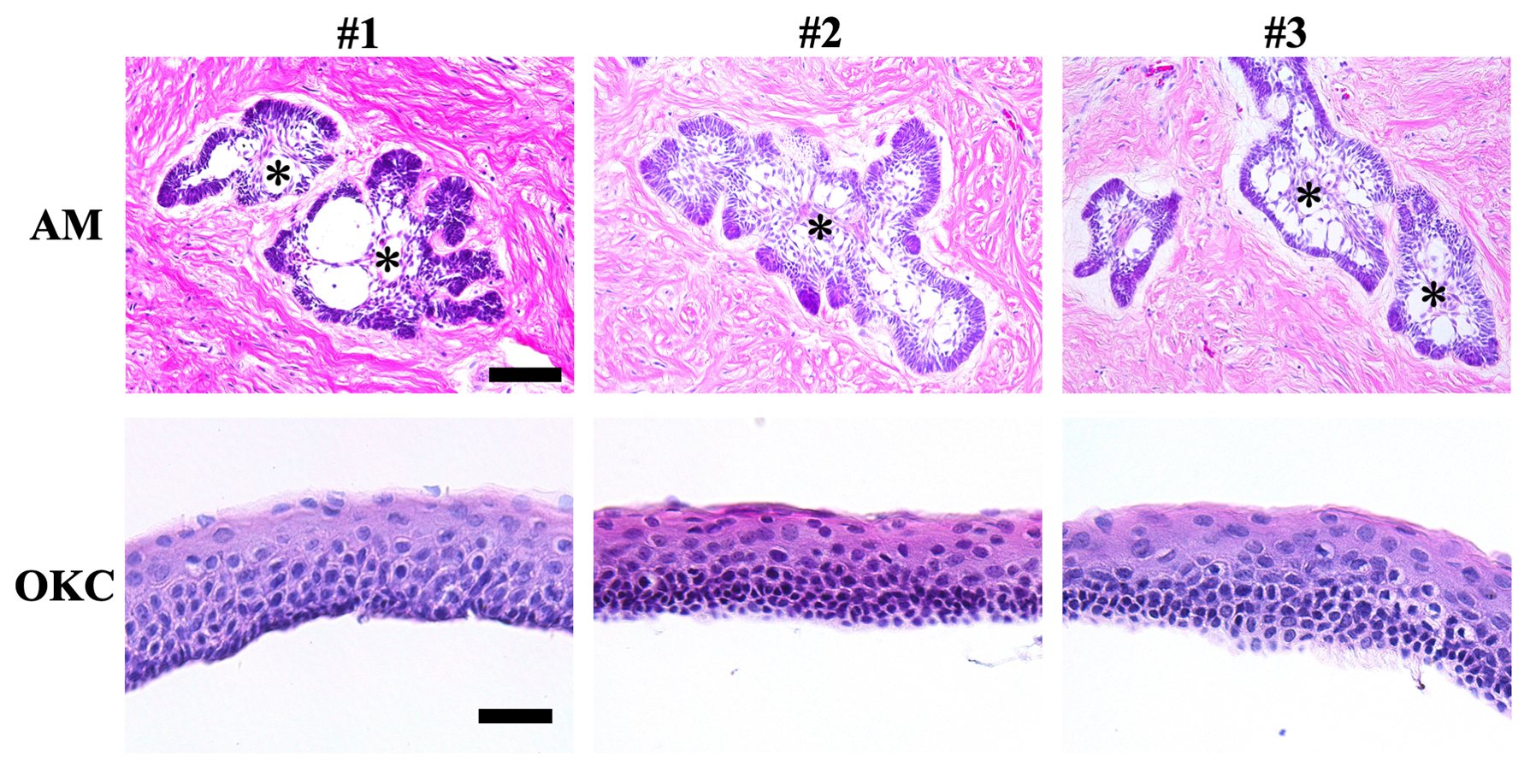


**Fig. S2 | Histopathological validation for the AM and OKC samples.**

The Hematoxylin and Eosin staining images of the three AM and three OKC samples, which were used for RNA-sequencing. Three AM samples were diagnosed to a follicular variant of the conventional ameloblastoma. The islands of epithelium have a peripheral layer of palisaded, elongated basal cells with a suprabasal ‘stellate reticulum’ zone (*). Three OKC samples were characterized by a uniform para-keratinized epithelial lining with prominent, often columnar, basal cells showing reversed polarity, as commonly seen in epithelial odontogenic tumors. Scale bar: AM, 100 μm; OKC, 50 μm.


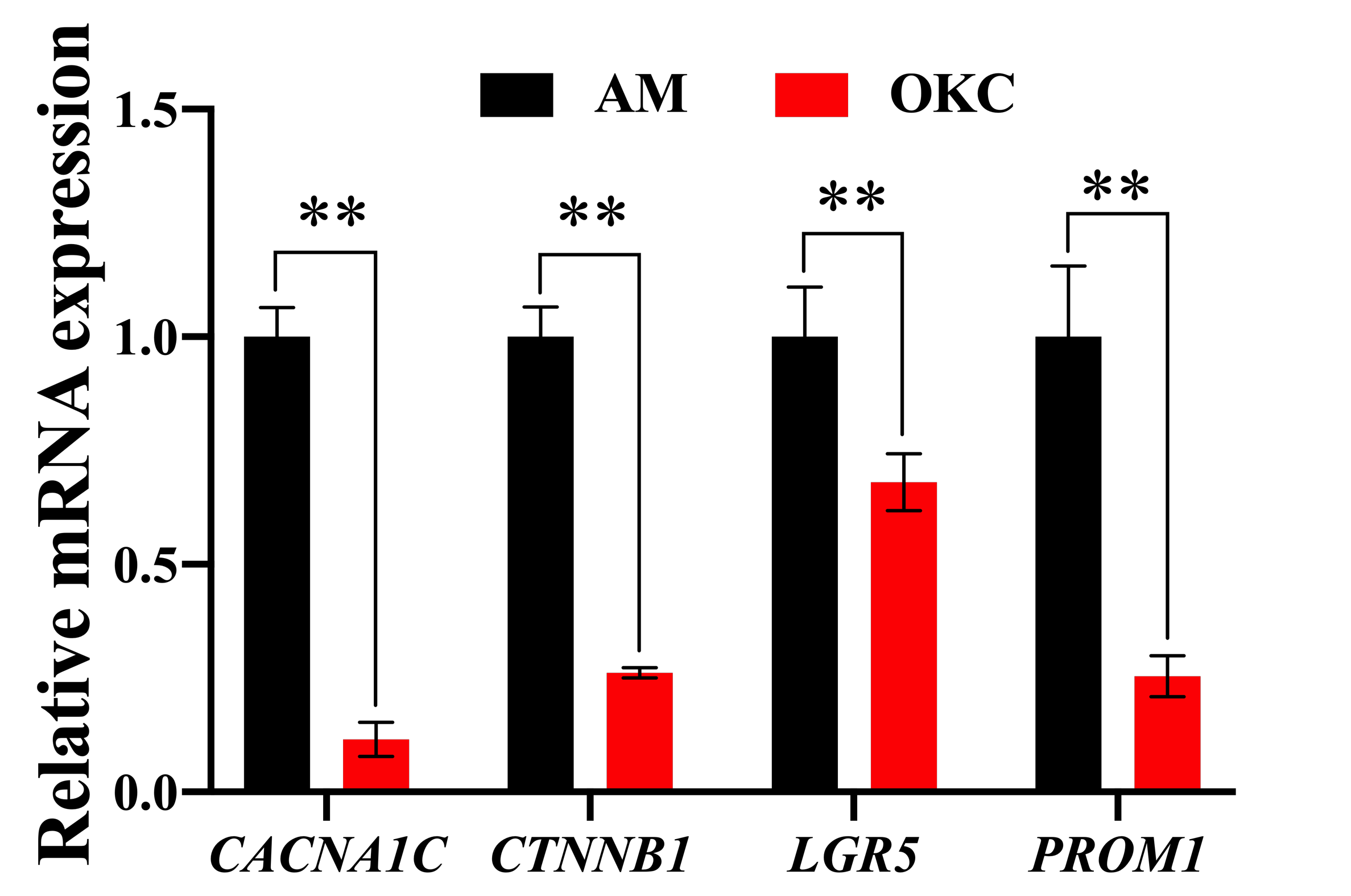


**Fig. S3 | Validation for the mRNA expression of selected parameters from RNA-sequencing.**

The relative mRNA expression of CACNA1C, CTNNB1, LGR5 were significantly upregulated in AM compared to the OKC samples (n=5). Quantitative data are presented as the mean ± SD. *p*-value: ***p* < 0.01.

**Fig. S4 | Cytotoxicity of verapamil.**

Dose-response curve of AM cells to verapamil for 16h determined by the CCK-8® cytotoxicity assay. Assays were performed according to the kit (CK04-01, Dojindo) protocol in triplicate. The determined IC_50_ is 100.8 μM.


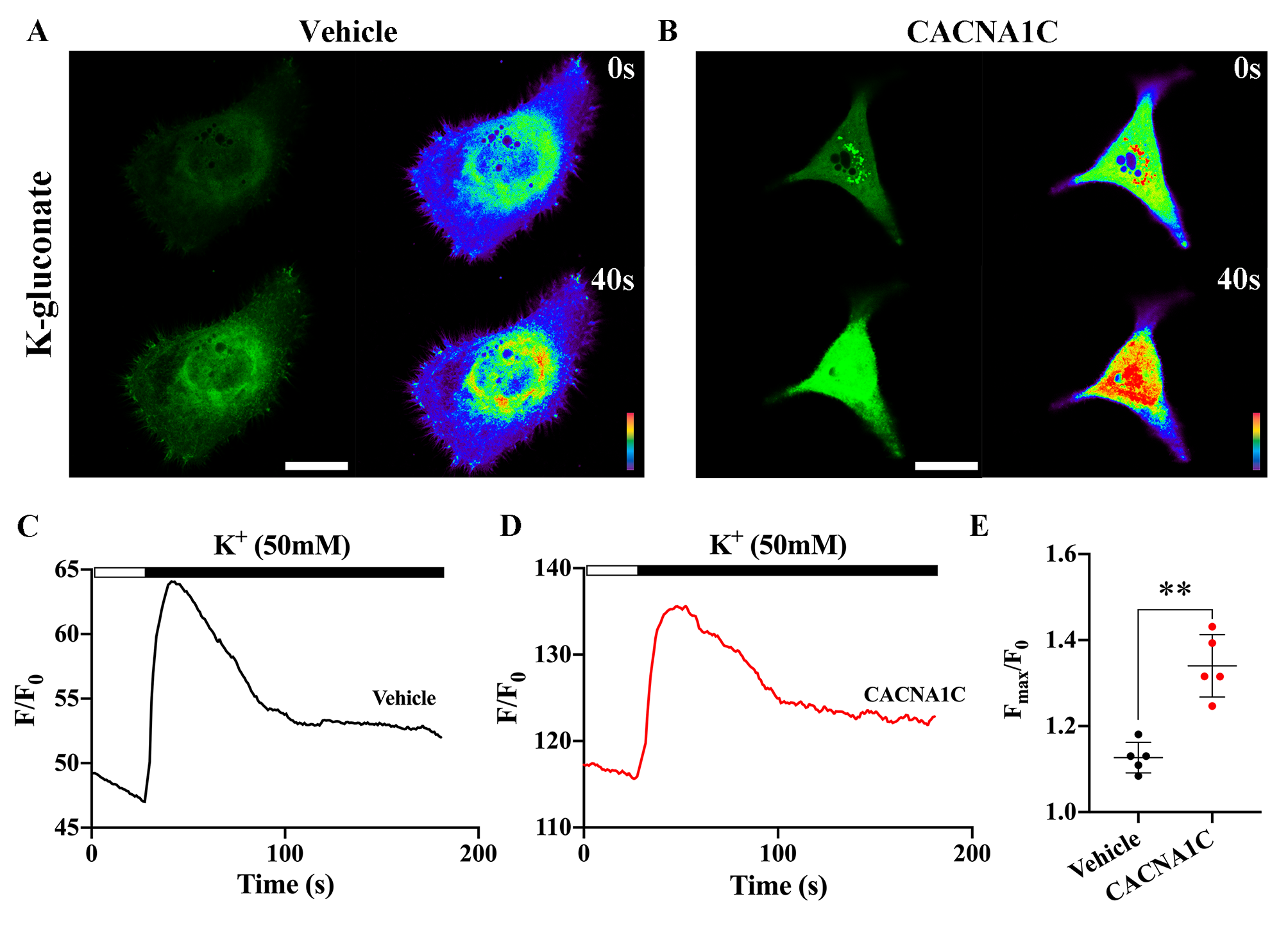


**Fig. S5 | Ca^2+^ influx increased in CACNA1C-overexpressed AM cells.**

(A, B) Confocal imaging of Ca^2+^ elevation in vehicle and CACNA1C-overexpressed AM cells. The Ca^2+^ transients are indicated with Fluo-4 AM (green), and the depolarization was induced by K-gluconate (50 mM) solutions. Calcium imaging was obtained at 1s intervals for a total duration of 4 min. Scale bar: 25$\mu$m. (C, D) Fluo-4 imaging of the Ca^2+^ response to K-gluconate in vehicle and CACNA1C-overexpressed AM cells at each time point. Note that the horizontal bar indicates the time of K-gluconate addition. The raw data are expressed as F/F_0_ (F: fluorescence intensity; F_0:_ mean fluorescence intensity before stimulation). (E) F_max_/F_0_ (F_max_: Maximum fluorescence intensity upon stimulation) ratios in the Cav1.2-overexpressed group was significantly higher than vehicle. Quantitative data are presented as the mean ± SD. *p*-value: ***p* < 0.01.

**
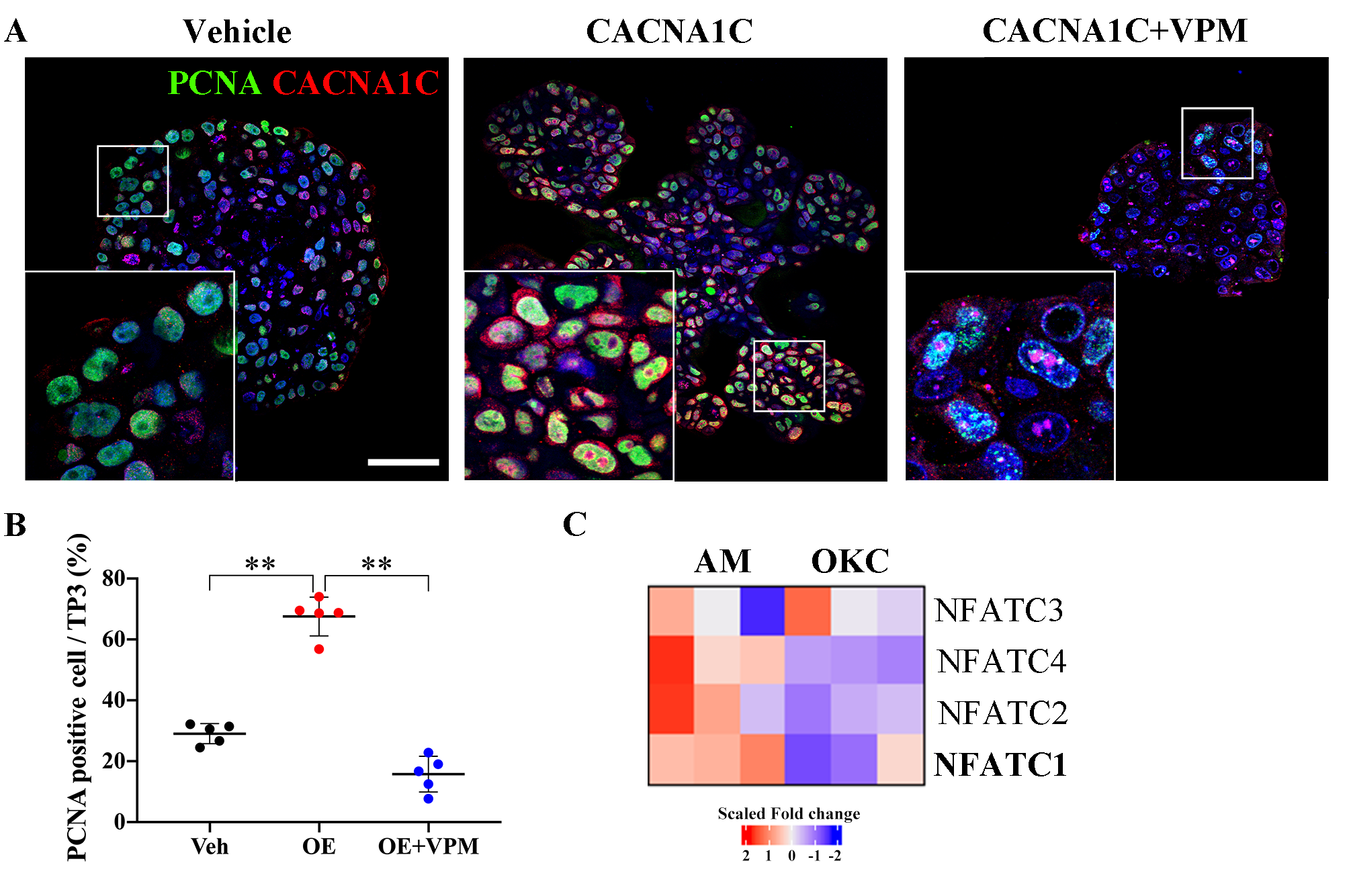
**

**Fig. S6 | The cell proliferation strongly associated with the Cav1.2 in AM tumoroid.**

(A) Representative confocal image of PCNA and CACNA1C staining in vehicle, CACNA1C-overexpression and VPM treatment AM tumoroids. PCNA positive AM cells were localized in peripheral region or budding region of AM tumoroid in vehicle and CACNA1C group respectively. And it negatively expressed in VPM treatment group. Nuclear were stained with TP3 (blue). Scale bar: 50 μm. (B) Quantification of PCNA positive cell number. PCNA positive AM cells were significantly increased in CACNA1C overexpression group compared to vehicle and VPM treatment group. (C) Heatmap of NFAT family gene expression between AM and OKC. Quantitative data are presented as the mean ± SD. *p*-value: ***p* < 0.01. Unmarked significance assessment means not significant.

**
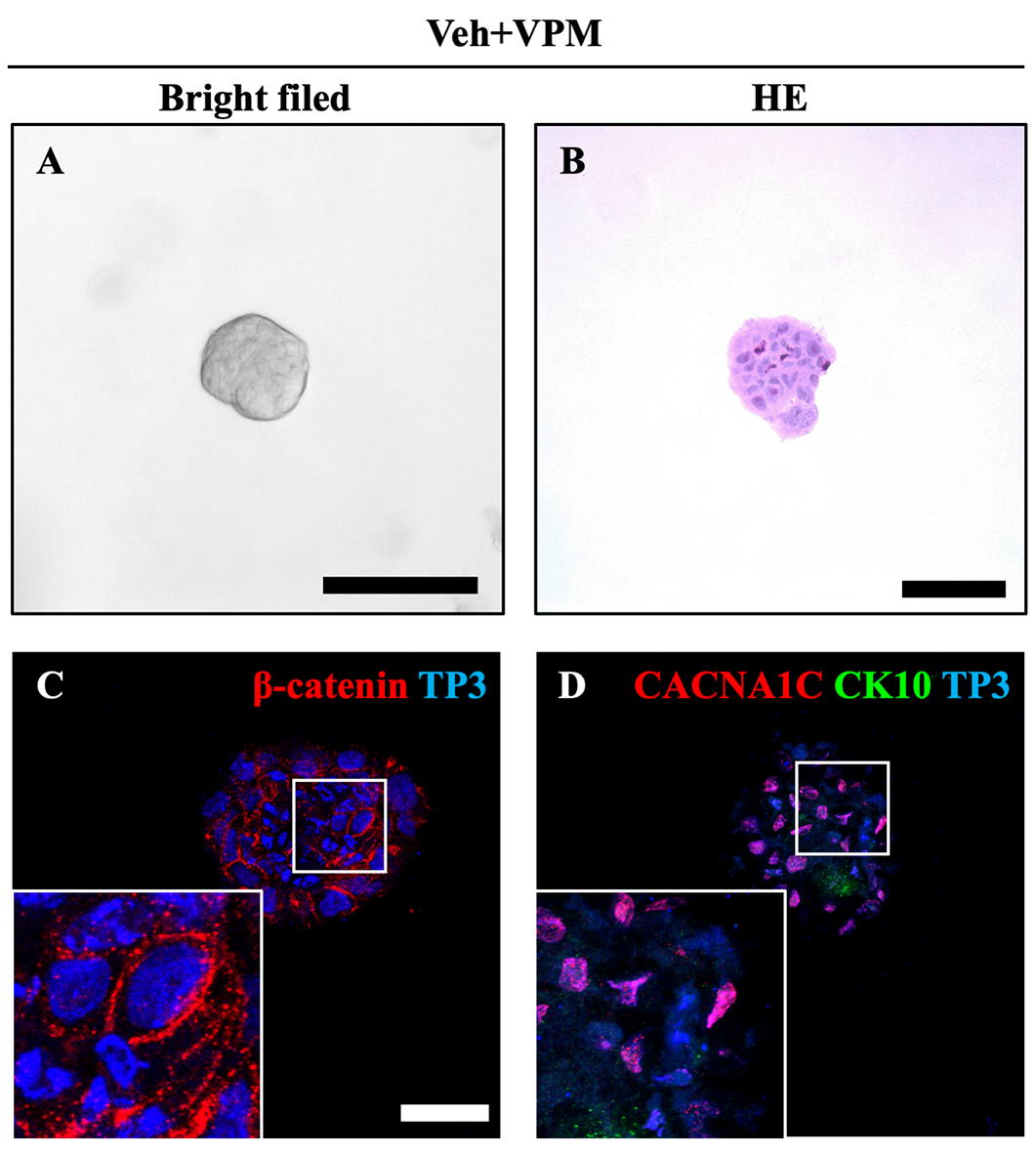
**

**Fig. S7 | Cav1.2 maintained Wnt/ β-catenin signaling activity in AM tumoroid.**

(A, B) Representative bright filed image and Hematoxylin and Eosin staining of AM tumoroid in the vehicle group with the presence of VPM (10 μM). (C) Nuclear translocated β-catenin is not observed in the AM tumoroid. (D) CK10 expresses faintly in part of AM tumoroid. Scale bar: A, 300 μm; B, 100 μm; C, D, 50 μm.
